# Low cholesterol efflux capacity and abnormal lipoprotein particles in youth with type 1 diabetes: a case control study

**DOI:** 10.1186/s12933-018-0802-0

**Published:** 2018-12-19

**Authors:** Evgenia Gourgari, Martin P. Playford, Umberto Campia, Amit K. Dey, Fran Cogen, Stephanie Gubb-Weiser, Mihriye Mete, Sameer Desale, Maureen Sampson, Allen Taylor, Kristina I. Rother, Alan T. Remaley, Nehal N. Mehta

**Affiliations:** 1 Division of Pediatric Endocrinology, Department of Pediatrics, Georgetown University, 4200 Wisconsin Avenue, N.W, 4th Floor, Washington, DC 20016 USA; 2 Section of Inflammation and Cardiometabolic Diseases, National Heart, Lung, and Blood Institute, National Institutes of Health, Bethesda, MD USA; 3 Cardiovascular Medicine, Brigham and Women’s Hospital, Harvard Medical School, Boston, MA USA; 4 Division of Pediatric Endocrinology, Department of Pediatrics, Children’s National Health Systems, George Washington University, Washington, DC USA; 5 Clinical Research Unit, Georgetown University, Washington, DC USA; 6 Department of Biostatistics and Biomedical Informatics, MedStar Health Research Institute, Hyattsville, MD USA; 7 Section of Lipoprotein Metabolism, National Heart, Lung and Blood Institute, National Institutes of Health, Bethesda, MD USA; 8 Division of Cardiology, Georgetown University School of Medicine, Washington, DC USA; 9 Section on Pediatric Diabetes and Metabolism, National Institute of Diabetes and Digestive and Kidney Diseases, National Institutes of Health, Bethesda, MD USA

**Keywords:** Type 1 diabetes, Cholesterol efflux, NMR, Adolescent, Cardiovascular risk

## Abstract

**Background:**

Patients with type 1 diabetes (T1DM) have increased mortality from cardiovascular disease (CVD). Risk factors for CVD include an elevation of LDL (LDLp) and small HDL (sHDLp) particles, and a decrease in reverse cholesterol transport i.e. HDL-cholesterol efflux capacity (CEC). Our objective was to compare lipoprotein particles and CEC between T1DM and healthy controls (HC) and to explore the associations between NMR lipid particles and cholesterol efflux.

**Methods:**

78 patients with T1DM and 59 HC underwent fasting lipoprotein profile testing by NMR and measurements of CEC by cell-based method. The associations between NMR lipid particles with CEC were analyzed using multivariable linear regression models.

**Results:**

Youth with T1DM had higher total LDLp 724 [(563–985) vs 622 (476–794) nmol/L (P = 0.011)] (Maahs et al. in Circulation 130(17):1532–58, 2014; Shah et al. in Pediatr Diabetes 16(5):367–74, 2015), sHDLp [11.20 (5.7–15.3) vs 7.0 (3.2–13.1) μmol/L (P = 0.021)], and lower medium HDLp [11.20 (8.5–14.5) vs 12.3 (9–19.4), (P = 0.049)] and lower CEC (0.98 ± 0.11% vs 1.05 ± 0.15%, P = 0.003) compared to HC. Moreover, CEC correlated with sHDLp (β = − 0.28, P = 0.045) and large HDLp (β = 0.46, P < 0.001) independent of age, sex, ethnicity, BMIz, HbA1c, hsCRP and total HDLp in the diabetic cohort.

**Conclusions:**

Youth with T1DM demonstrated a more atherogenic profile including higher sHDL and LDLp and lower CEC. Future efforts should focus on considering adding lipoprotein particles and CEC in CVD risk stratification of youth with T1DM.

*Trial registration* Clinical Trials Registration Number NCT02275091

## Background

Patients with type 1 diabetes (T1DM) are at increased risk of cardiovascular disease (CVD) [1]. The CVD associated processes starts early in childhood. It is well established that youth with T1DM exhibit early signs of CVD, such as higher blood pressure, arterial stiffness, myocardial and endothelial dysfunction [1–4]. Some of the known risk factors include an abnormal lipoprotein profile, characterized by small Low-density lipoprotein (LDL) particles (LDLp) and small High-density lipoprotein (HDL) particles (HDLp) and a decrease in reverse cholesterol transport i.e. low HDL-cholesterol efflux capacity (CEC) [1, 5].

Atherogenic lipoprotein profiles based on traditional lipid panel are characterized by high LDL cholesterol and low HDL cholesterol. A more detailed lipid characterization can be obtained with the use of nuclear magnetic resonance (NMR) profiling [6, 7]. Evidence of total LDL particles (LDLp), small size LDL particles (sLDLp) and small size HDL particles (sHDLp) is suggestive of a more atherogenic and less cardio-protective profile [8–12]. Patients with T1DM often have a favorable HDL cholesterol, measured with traditional assays, compared to healthy subjects, which seems to suggest that HDL cholesterol, which is typically protective against CVD, is not a major factor influencing CVD risk in T1DM [13]. Furthermore, the failure of therapies that increase HDL cholesterol concentration to improve cardiovascular outcomes has led to new research that investigates HDL function, rather than HDL concentration [14]. Cholesterol efflux capacity (CEC) is the ability of HDL-C to promote reverse cholesterol transfer from macrophages to the liver and plays an important role in decreasing the development of atherosclerosis [5, 14]. Low CEC has been linked to cardiovascular events in a cohort of 2924 healthy volunteers that were followed for 9.4 years and to angiographic evidence of coronary disease and carotid intima media thickness suggesting it is a strong marker of increased CVD risk [5, 15].

Young patients with T1DM may have normal or subtle changes in lipid profiles not detected on routine lab testing. Our objective was to compare NMR lipoprotein particles and cholesterol efflux between T1DM and healthy controls (HC) and to explore the associations between NMR lipid particles with cholesterol efflux. We hypothesized that T1D would have increased smaller lipid particles (in particular small HDL-p) and impaired efflux capacity.

## Methods

All patients were enrolled in the clinical protocol “Identifying Children with type 1 diabetes at high risk for CVD” (Clinical Trials Number: NCT02275091) that was approved by the Georgetown-Howard Universities Center for Clinical and Translational Science (GHUCCTS) Institutional Review Board (IRB). All adult patients and all parents of pediatric patients provided written informed consent and all children signed an assent.

Children and young adults with T1DM between the ages of 12 and 21 years were eligible for participation. Subjects on lipid lowering medications were excluded. Healthy individuals between the ages of 12 and 21 years were also eligible for participation. Special attention was paid that the participants were not taking any lipid lowering medications.

Recruitment of study participants was done by sending IRB approved letters to the patients with T1DM who are followed at the Pediatric Endocrine Divisions of Georgetown University (GU), Howard University (HU) and Children’s National Health System (CNHS) in Washington, DC. Healthy controls were recruited using the Research Match database, as well as by sending flyers to local pediatricians and letters to families of children who had their well-child visits in the pediatric clinic of GU.

Biochemical evaluation was done at the core lab at GU after a 10 h overnight fast. hsCRP and HbA1c was measured using the Siemens Dimension VISTA system. The HbA1c measurement is based on a turbidimetric inhibition immunoassay principle, and the measurement of total hemoglobin is based on a modification of the alkaline hematin reaction. The HbA1C testing we performed is an FDA validated and approved methodology. Our test was thoroughly validated and approved by our lab prior to implementing the test platform. The core lab also performs daily quality control testing, calibrates as required by the manufacturer, and performs blinded proficiency test samples from an outside agency. Using the values obtained for each of these two analytes (in g/dL), the percentage of the total hemoglobin that is glycated is calculated and reported as % HbA1c. Research blood was processed and stored in − 80 °C until assay performance at the NIH.

Carotid Intima Media Thickness (CIMT) was measured by two expert cardiologists at GU, using a Sonosite ECHO machine. The CIMT was measured at the common carotid artery, bulb and at the right and left side. Two measurements were done at each location, the maximum CIMT and the minimum CIMT and the average of all measurements was used to calculate the mean average CIMT and the mean maximum CIMT.

A research nurse conducted the anthropometric measurements. The Body Mass Index (BMI) z score was calculated using the CDC charts. Waist circumference was measured at the minimum perimeter between the iliac crest and the rib cage. Waist measurements were done three times and averaged. Ethnicity and total units of insulin used were self-reported.

Lipoprotein particle number, size and concentration were measured using Liposcience NMR spectroscopy at the National Institutes of Health Clinical Center as previously described [16]. HDLp [total, small(s), medium (m) and large (l)] is reported in μmol/L. LDLp and VLDLp (all sizes) are reported in nmol/L. In referring to different parameters, the following suffixes are used: z = size, p = particle number, c = cholesterol concentration. Cholesterol is measured in mg/dL; triglyceride concentration is abbreviated as tg and expressed as mg/dL. Apolipoprotein AI and Apolipoprotein B were derived from Liposcience NMR.

HDL cholesterol efflux capacity assays were performed based on published methods, using the murine macrophage cell line, J774 [5, 15, 17]. Briefly, 3 × 10^5^ J774 cells/well were plated and radiolabeled with 2 µCi of ^3^H-cholesterol/mL for 24-h. ATP-binding cassette transporter A1 (ABCA1) was up-regulated by means of a 16-h incubation with 0.3 mmol/L 8-(4-chlorophenylthio)-cAMP. ApoB-depleted plasma (2.8%) was added to the efflux medium for 4 h. Liquid scintillation counting was added to quantify the efflux of radioactive cholesterol from the cells. Efflux was calculated by using the following formula: (µCi of ^3^H-cholesterol in media containing 2.8% apoB-depleted subject plasma-µCi of ^3^H-cholesterol in plasma-free media/µCi of ^3^H-cholesterol in media containing 2.8% apoB-depleted pooled control plasma-µCi of ^3^H-cholesterol in pooled control plasma-free media). The pooled plasma was obtained from five healthy adult volunteers. All assays were performed in at least duplicates. We also described the characteristics of youth with T1DM that had low CEC. We defined low CEC as the values below the median CEC in the T1DM group.

### Statistical methods

Data were summarized using means and standard deviation (SD) for normally distributed variables, medians and 25–75th quartile for non-normally distributed variables, and frequencies and percentages for categorical variables. Two-sample *t* test was used to compare continuous variables, and Fisher’s exact test to compare categorical variables by study groups. Pearson’s correlation coefficient was used to assess linear correlations. Analyses were also carried out to examine the differences between the “low efflux” (CEC ≤ median) and “high efflux” (CEC > median) subgroups of patients with T1DM. We chose to use as cut-off the median value due to the lack of robust data on physiologic normal thresholds for cholesterol efflux in youth with T1DM. Regression analyses were conducted to analyze the relationships between CEC and the different sizes of HDL particles adjusting for age, sex, ethnicity, BMI-z score, hsCRP, HbA1c and total HDLp. A *P* value of < 0.05 was considered statistically significant. Data were analyzed using STATA version 15.

## Results

### Subjects characteristics

We enrolled adolescents and young adults with T1DM (n = 78) and healthy controls (n = 59) over a 3-year period. Patients with T1DM had a mean age of 16.7 ± 1.8 years, 57.7% were females, and 74% were Caucasian. Median diabetes duration was 3.0 (2.0–4.0) years. Mean patient HbA1c% was 8.4 ± 1.4% and the mean BMI z score was 0.46 ± 0.86. The healthy controls had a mean age of 17.3 ± 2.4 years, 47.5% were females, 44% were Caucasian. Mean HbA1c% for healthy controls was 5.3 ± 0.4%, and the mean BMI z score was − 0.06 ± 1.06. The demographic characteristics of patients with T1DM and HC and the laboratory results are also shown on Table 1. Because of the observed differences in BMI-z score and ethnicity, we also calculated the P value of main outcomes adjusted for these differences.

**Table 1:** Characteristics of study group for adolescents with T1DM and healthy controls

Variable	Diabetes (N = 78)	Controls (N = 59)	P-value	Adjusted P-value
*Demographic and clinical characteristics*				
Age, years	16.7 ± 1.8	17.3 ± 2.4	0.090	
Sex female (%)	45 (57.7%)	28 (47.5)	0.234	
Body mass index, kg/m^2^	23.3 ± 3.7	21.8 ± 3.8	*0.020*	
BMI z score	0.46 ± 0.86	− 0.06 ± 1.06	*0.002*	
Ethnicity, n = Caucasian (%)	58 (74%)	26 (44%)	*<* *0.001*	
Diabetes duration, years	3 (2.0–4.0)	–		
Insulin dose (units/kg/day)	0.81 ± 0.32	–		
Carotid intima media thickness mean (mm)	0.478 ± 0.057	0.472 ± 0.054	0.530	0.964
Carotid intima media thickness max (mm)	0.538 ± 0.073	0.525 ± 0.063	0.297	0.942
*Clinical and lab values*				
SBP	111 ± 11	111 ± 10	0.717	0.411
SBP z score	− 0.29 ± 0.94	− 0.19 ± 0.86	0.490	0.295
DBP	62 ± 8	63 ± 8	0.357	0.365
DBP z score	− 0.42 ± 0.69	− 0.37 ± 0.81	0.695	0.273
Total cholesterol, mg/dL	142.2 ± 26.1	133.9 ± 23.1	0.060	*0.046*
HDL cholesterol, mg/dL	56.9 ± 10.2	57.9 ± 10.6	0.592	0.448
LDL cholesterol, mg/dL	74.6 ± 24.8	66.3 ± 22.2	0.051	*0.041*
Triglycerides, mg/dL	83.4 ± 33.3	81.4 ± 30.9	0.726	0.885
High sensitive C-reactive protein, mg/L	0.6 (0.25–1.45)	0.3 (0.2–0.8)	*0.019*	0.074
HDL efflux capacity	0.98 ± 0.11	1.05 ± 0.15	*0.003*	*0.038*
HbA1c	8.40 ± 1.35	5.33 ± 0.43	*<* *0.001*	*<* *0.001*
*NMR values*				
HDL particle number	30.50 (27.7–33.9)	30.6 (29.1–32.7)	0.755	0.364
Small HDL particle number	11.20 (5.7–15.3)	7.0 (3.2–13.1)	*0.021*	*0.026*
Medium HDL particle number	11.20 (8.5–14.5)	12.3 (9–19.4)	*0.049*	*0.013*
Large HDL particle number	7.8 (6.1–9.7)	8.4 (6.3–10.4)	0.235	0.433
HDL-z	9.9 (9.5–10.2)	9.9 (9.6–10.3)	0.314	0.373
LDL particle number	724 (563–985)	622 (476–794)	*0.011*	*0.017*
Small LDL particle number	300 (0.1–431)	242 (0.1–343)	0.053	0.078
Large LDL particle number	259 (80–404)	197 (54–359)	0.243	0.129
LDL-z	20.6 (20.1–21.1)	20.7 (20.1–21.2)	0.510	0.931
VLDL particle number	40.5 (29.5–49.1)	35.5 (29.9–46.5)	0.163	0.299
Small VLDL particle number	31.1 (23.6–37.5)	27 (15.2–32.4)	*0.023*	*0.028*
Medium VLDL particle number	8.3 (3.9–13.2)	9.3 (4.5–12.2)	0.710	0.295
Large VLDL particle number	1.8 (1.1–3.1)	2.2 (1.2–4.5)	0.187	0.296
VLDL z	46.5 (43.9–49.2)	49.5 (45.2–53.2)	*0.005*	*0.007*
Apolipoprotein A	141.6 (129.7–157.3)	143.1 (128.6–157.2)	0.802	0.963
Apolipoprotein B	75.9 (63.7–83.4)	62.8 (53.3–74.3)	*0.001*	*0.001*
Ratio of Apo A/Apo B	2.03 (1.59–2.32)	2.29 (1.92–2.69)	*0.004*	*0.004*

All values are expressed as mean ± SD or median (25–75th percentile). P-value is calculated between groups for variables using unpaired t-test for continuous variables, Pearson’s Chi squared test for categorical variables and Rank sum for non-parametric tests. Adjusted P values show the significance level of the differences based on robust regression models adjusted for ethnicity and BMI z score. z is reported as nanometer; Tg and cholesterol are reported as milligrams per deciliter, HDLp (total, small, medium and large) are reported as micromoles per liter, whereas LDLp and VLDLp (all sizes) are reported as nanomoles per liter

Italic values indicate significance of P value (P < 0.05 )

### Comparison of lipoproteins, CEC and CIMT in T1DM versus HC

There were no significant differences in total cholesterol, total HDL cholesterol and triglycerides between T1D and healthy participants, LDL being 8 mg/dL higher in T1DM compared to controls (Table 1). In patients with T1DM, median Apolipoprotein B was significantly higher [(75.9 (63.7–83.4) vs 62.8 (53.3–74.3) mg/dL, P = 0.001] and the median Apolipoprotein A-I/Apolipoprotein B ratio was significantly lower [2.03 (1.59–2.32) vs 2.29 (1.92–2.69), P = 0.004] compared to HC.

NMR lipoprotein profiles in patients with T1DM demonstrated higher total LDLp, small HDLp and small VLDLp and lower medium HDLp when compared to HC (Table 1). There were no differences in total HDLp, large HDLp and total, medium and large VLDLp (Table 1).

In individuals with T1DM, HDL cholesterol efflux capacity (CEC) was significantly lower than in controls (CEC 0.98 ± 0.11% vs 1.05 ± 0.15%, P = 0.003).

Measurement of carotid intima media thickness did not reveal differences between the T1DM patients and healthy controls (Table 1). While not significant in the individual cohorts, both small LDLp and small HDLp correlated with maximum CIMT (r = 0.18, P = 0.048 and r = 0.23, P = 0.01 respectively) in the entire study cohort.

### Comparison of “low CEC” and “high CEC” groups in subjects with T1DM

When the subjects with T1DM were dichotomized in two separate groups by median CEC capacity, the low CEC group was different when compared to high CEC group in terms of having lower total HDL cholesterol, lower large HDLp and lower HDL-z, lower Apolipoprotein A, lower ratio of Apolipoprotein A/Apolipoprotein B and higher total LDLp and small LDLp (Table 2).

**Table 2:** Characteristics of T1DM subgroups with low and high efflux

Variable	Diabetes low efflux n = 37	Diabetes high efflux n = 37	P-value
*Demographic and clinical characteristics*			
Age, years	17.0 ± 1.6	16.5 ± 1.9	0.191
Sex female (%)	21 (57)	21 (57)	1.000
Body mass index, kg/m^2^	23.5 ± 4.0	23.3 ± 3.6	0.804
BMI z score	0.42 ± 0.95	0.51 ± 0.80	0.644
Ethnicity, n-Caucasian (%)	26 (70)	28 (76)	0.397
Diabetes duration, years	3 (1–13.5–8)	3 (1–5)	0.200
Carotid intima media thickness mean	0.486 ± 0.070	0.474 ± 0.045	0.386
*Clinical and lab values*			
SBP	111 ± 10	110 ± 13	0.634
SBP z score	− 0.29 ± 0.80	− 0.28 ± 1.1	0.935
DBP	62 ± 7	62 ± 9	0.953
DBP z score	− 0.46 ± 0.62	− 0.38 ± 0.78	0.647
Total cholesterol, mg/dL	141.9 ± 26.6	142.0 ± 26.1	0.999
HDL cholesterol, mg/dL	52.8 ± 7.9	60.4 ± 10.7	*0.001*
LDL cholesterol, mg/dL	78.7 ± 26.2	70.4 ± 23.9	0.159
Triglycerides, mg/dL	86.1 ± 37.1	82.1 ± 30.4	0.609
High sensitive C-reactive protein, mg/dL	0.55 (0.2–15.8)	0.60 (0.2–9.5)	0.593
HDL efflux capacity	0.89 ± 0.07	1.07 ± 0.07	*<* *0.001*
HbA1c	8.26 ± 1.22	8.50 ± 1.49	0.458
*NMR values*			
HDL particle number	29.8 (23.4–45.2)	31.1 (24.2–42.2)	0.155
Small HDL particle number	12.5 (0–25.8)	10.0 (0.1–20.8)	0.375
Medium HDL particle number	10.8 (2.0–28.3)	11.8 (2.227.9)	0.593
Large HDL particle number	6.5 (3.3–10.0)	9.2 (3.9–17.4)	*0.0003*
HDL-z	9.7 (8.7–10.7)	10.0 (8.9–10.7)	*0.018*
LDL particle number	811 (316–1553)	713 (90–1171)	*0.054*
Small LDL particle number	342 (0–1236)	218 (0.0–947)	*0.006*
Large LDL particle number	252 (0.1–709)	279 (0–650)	0.619
LDL-z	20.5 (19.7–21.8)	20.7 (19.6–21.6)	0.284
VLDL particle number	40.3 (0–85)	40.6 (12.0–91.7)	0.957
Small VLDL particle number	34.0 (0.5–57.1)	31.1 (5.3–67.8)	0.829
Medium VLDL particle number	7.1 (0–37.5)	9.4 (0.1–33.4)	0.430
Large VLDL particle number	2.2 (0.114.0)	1.8 (0.2–13.7)	0.183
VLDL z	47.0 (36.5–63.2)	46.5 (40.0–71.2)	0.631
Apolipoprotein A	135.0 (102.5–181.4)	154.3 (125.8–237.2)	*0.0001*
Apolipoprotein B	75.9 (36.0–137.9)	75.9 (27.1–121.1)	0.905
Ratio of Apo A/Apo B	1.85 (0.92–3.63)	2.09 (1.10–5.74)	*0.029*

All values are expressed as mean ± SD or median (25–75th percentile). P-value is calculated between groups for variables using unpaired t-test for continuous variables, Pearson’s Chi squared test for categorical variables and Rank sum for non-parametric tests. Tg and cholesterol are reported as milligrams per deciliter, HDLp (total, small, medium and large) are reported as micromoles per liter, whereas LDLp and VLDLp (all sizes) are reported as nanomoles per liter

Italic values indicate significance of P value (P < 0.05 )

### Correlations of CEC with HDL particles in T1DM and in HC groups

The CEC correlated positively with large HDLp (r = 0.40, P = 0.0004) and negatively with small HDLp (r = − 0.20, P = 0.09) in the T1DM group. The CEC correlated positively with large HDLp (r = 0.35, P = 0.01) and negatively with small HDLp (r = − 0.13, P = 0.35) in the HC group.

### Regressions of CEC with HDL particles in T1DM and in HC groups

**Fig. 1:**
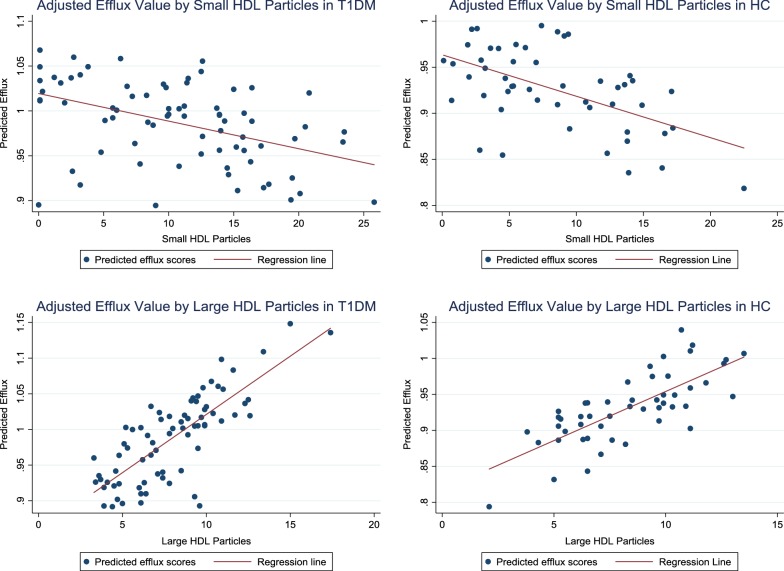
Predicted scores for CEC based on adjusted regression models and their relationships to small and large HDL particles

Linear regression models adjusted for age, sex, ethnicity, BMIz, HbA1c, hsCRP and total HDLp showed that CEC in T1DM was negatively associated with small HDLp (β = − 0.28, P = 0.045, model R2 = 0.17) and positively associated with large HDLp (β = 0.46, P < 0.001, model R2 = 0.28). CEC in healthy controls was not significantly associated with small HDLp (beta = − 0.06, P = 0.721, model R2 = 0.28) or with large HDLp (beta = 0.33, P = 0.08, model R2 = 0.33), after adjusting for age, sex, ethnicity, BMIz, HbA1c, hsCRP and total HDLp. Predicted scores for CEC based on adjusted regression models and their relationships to small and large HDL particles are presented in Fig. 1 for each group.

## Discussion

In our observational cohort of youth with T1DM and healthy controls, we demonstrate important findings related to potential links between lipoprotein dysfunction and cardiovascular risk in T1DM. Youth with T1DM demonstrated a more atherogenic profile including higher small HDL particles and LDL particles and lower cholesterol efflux capacity compared to healthy controls.

Firstly, using the traditional lipid panel, we found no differences in total HDL-C between youth with T1DM and healthy controls, which is in agreement with multiple other studies showing that adult and pediatric patients with T1DM often have no differences in the total HDL-C or they might have a more favorable profile with higher HDL-C compared to HC [13, 18–20]. However, we were able to show differences using NMR, which provides a more detailed lipoprotein profile than standard lipid analyses and can detect abnormalities on the HDL cholesterol that can suggest decreased HDL-efflux and can also further characterize the LDL-C abnormalities. One study with 194 adults with T1DM using NMR found that women with T1DM had higher small LDLp and lower large LDLp compared to non-diabetic women, but no differences were seen in LDLp (small and large) between men with T1DM and non-diabetic men [21]. Contrary to our findings, their HDL particle distribution, showed lower sHDLp and higher large HDLp compared to nondiabetic women and men, suggesting a favorable HDLp distribution profile in patients with T1DM [21]. One explanation for this difference could be that many subjects that participated in this study (age 30–55 years old) had previous heart disease and most likely were taking statins or other medications that could have affected their lipoprotein profile. Another possible explanation for this difference could be the factor of age, since our group of adolescents and young adults have unique characteristics, such as increased insulin resistance due to pubertal hormones, that could at least partially affect the lipoprotein profile distribution. It is also possible that high requirements for insulin in adolescents compared to older adults with T1DM could also play a role in the different HDL particle distribution.

Another study examined the NMR lipoprotein profile in adults with T1DM from the DCCT/EDIC [6]. They found that patients treated with intensive insulin therapy, with an average HbA1c of 7.2%, had a favorable atherogenic profile with lower small LDLp and small HDLp compared to the conventionally treated group, with an average HbA1c of 9.1% [6]. This study suggests that some of the favorable clinical outcomes from the intensive insulin regimen can be attributed to the favorable LDL and HDL subclass characteristics. It also supports the notion that excellent glycemic control and an intensive insulin regimen are associated with favorable HDL and LDL subclasses distribution. Furthermore, two more studies from the DCCT/EDIC trial showed significant positive associations of large and small LDLp and an inverse association of large HDLp with CIMT supporting the use of NMR profile, in addition to the conventional lipid profile, to identify patients with T1DM at high risk for CVD [22, 23].

Secondly, we found that youth with T1DM have decreased cholesterol efflux compared to healthy controls. Cholesterol efflux has been associated with subclinical atherosclerosis as reflected in greater carotid intima media thickness among 203 healthy controls and patients in 442 patients with angiographically confirmed coronary artery disease [15]. Cholesterol efflux has also been inversely correlated with cardiovascular events in a population-based cohort of 2924 adult patients [5]. Previous studies in patients with diabetes have mainly focused on adults with type 2 diabetes (T2DM), with limited studies in adults and pediatric patients with T1DM. In agreement with our results, one recent study in pediatric patients with T1DM found lower cholesterol efflux, which was measured indirectly as a HDL-apoA–I exchange ratio, which reflects HDL function based on the concentration of Apo-AI [24]. We confirmed these results by using the gold standard method of measuring CEC which is the cell-based method and further looked into the relationship of CEC with HDL lipoprotein particles. Of note, the median duration of diabetes in our cohort was only 3 years, which suggests that the functional changes in HDL start very early in the course of the disease and might deteriorate over time even more.

Our study was not designed to offer a detailed mechanistic insight; however, we can only speculate about possible mechanisms that could explain the low CEC in youth with T1DM. One such explanation could be that youth with T1DM have altered HDL proteome that affects their CEC. It is known that several HDL proteins, such as apolipoprotein A, apolipoprotein D and apolipoprotein E are responsible for the CEC of HDL and their post-translational modification can impair the function of HDL [25, 26]. In support of this hypothesis and in agreement with our results, a recent study compared 30 adults with T1DM (both poorly and well controlled) with 30 non-diabetic controls and found decreased CEC in T1DM, irrespective of their glycemic control [25]. Interestingly the authors of this study found alterations in HDL-bound proteins that could partially explain the decreased CEC in this population [25]. Another possible explanation is that advanced glycation end products caused by T1DM impair HDL efflux, as it has been shown in some in vitro studies [26–28]. Future studies can better investigate the mechanism that can explain the low CEC in patients with T1DM.

Contrary to our findings, an older study among 14 adults with T1DM and average HbA1c 7.8 ± 1.3% found that patients with T1DM had higher efflux compared to healthy controls [29]. Possible explanations for this discrepancy are the different methods used to measure CEC and the different population characteristics seen in adolescents vs adults, such as higher insulin resistance of puberty that might affect HDL particles size and indirectly the CEC. One study among 35 adults with T2DM showed that they had decreased CEC compared to healthy controls, not only measured in the plasma but also in the interstitial fluid [30]. On the contrary, another study found enhanced cholesterol efflux among 45 patients with T2DM and hypertriglyceridemia compared to 26 patients with T2DM without hypertriglyceridemia, suggesting that high triglycerides in T2DM might produce a compensatory effect to maintain ABCA-1 efflux, although the mechanism is not entirely clear [31].

Several studies have tried to better characterize CVD risk in patients with T1DM [32, 33]. One of them showed that combining non-HDL cholesterol and apolipoprotein B provides a better estimation of increased CVD risk in adults with T1DM than any of these values used alone [32]. These results suggest that additional markers of CVD risk maybe complementary to each other and whether the addition of lipoprotein particles and CEC could further enhance the power of predictive models for CVD risk remains to be examined in future studies. Another very interesting study used data from the Swedish National Diabetes Register to better stratify CVD risk in adults with T1DM [33]. This study examined data from 33,333 patients with T1DM and compared them to controls without diabetes (5 healthy controls matched for every one patient with T1DM) randomly selected from the general population. This study showed a gradual increase in CVD risk for all-cause mortality, acute myocardial infarction, hospitalization for heart failure and stroke for every risk factor that was not at target level, such as HbA1c, blood pressure, low-density lipoprotein, albuminuria and smoking [33]. In this study, patients with T1DM who were well-controlled for all of the above risk factors, still showed increased CVD compared to the general population, suggesting that additional CVD risk markers, such as lipoprotein size and CEC, could further improve the CVD risk stratification in patients with T1DM.

Furthermore, we showed that cholesterol efflux in youth with T1DM is positively correlated with large HDLp and negatively with small HDLp after adjusting for age, sex, ethnicity, BMIz, HbA1c, hsCRP and total HDLp, while this relationship was not significant in the HC control. The relationship of CEC with the different sizes of HDL particles was investigated in a previous study of 44 patients with T2DM, and was significantly and positively correlated with total and medium size HDLp, but not with small HDLp [34]. However, there is some controversy about the relationship of HDLp size with CEC. Studies from in vitro experiments have shown that the small dense HDLp are the most important mediators of CEC via ABCA1 transporter, which is responsible for the CEC, and is found in more abundance in smaller dense HDLp [35]. More research is needed to clarify the relationship of cholesterol efflux and HDLp size and to determine whether interventions that increase large HDLp in youth with T1DM could lead to increased CEC.

One such possible intervention could be improving insulin sensitivity. A previous study by Nadeau et al. showed that low insulin sensitivity in youth with T1DM is associated with smaller size HDL and LDL lipoproteins using fast protein liquid chromatography (FPLC) [36]. Studies in adults with T1DM have also found that women with T1DM and insulin resistance have more atherogenic lipoprotein profile compared to non-diabetic women [37].

Finally, we showed that total HDL-C, large HDLp, HDL-z, small LDLp, Apolipoprotein A-I, and ratio of Apolipoprotein A/Apolipoprotein B are significantly different between the “low CEC” and “high CEC” subgroups of youth with T1DM. Given that measuring CEC is difficult in clinical practice compared to getting the results of NMR lipoproteins with a simple blood test, these values might be useful surrogate markers of decreased cholesterol efflux in youth with T1DM. Interestingly, glycemic control was not different in the two T1DM subgroups. Because our sample size was relatively small, larger studies will need to confirm these findings.

In our study, the observed difference in CIMT max (0.01 mm) between DM and HC groups was not statistically significant probably due to short diabetes duration and also our inadequate sample size to detect this difference. We are not able to make definite conclusions about the relationship of lipoprotein particles with CIMT, even though in the whole group both the small HDLp and the small LDLp correlated with CIMT. Other larger studies have previously found differences in CIMT between T1DM youth compared to HC [38]. Future studies can further investigate the association of lipoprotein particles in youth with T1DM and CEC with CIMT.

Strengths of our study include a well characterized cohort of youth with T1DM and healthy controls. We investigated for the first-time changes in cholesterol efflux using a direct assay with radiolabelled cholesterol and NMR lipoprotein profile in a young cohort of subjects with T1DM and explored their relationship with subclinical CVD. Limitations of our study include the relatively small size which may explain the inability to detect differences in CIMT between the youth with T1DM and healthy controls and therefore the lack of associations of small LDL and HDL with CIMT in the T1DM group only. However, we did find a correlation of small particles with CIMT in the whole group, and future studies should address this relationship again, also by looking at other more sensitive markers of subclinical CVD in youth with T1DM, such as arterial stiffness and endothelial dysfunction measures. Also, we did not have information about the exact Tanner stage of puberty in our participants; however, we believe the use of age maybe be a fair surrogate marker to assess for overall major differences in the puberty status that could affect the distribution of lipoproteins. Furthermore, our sample size was relatively small and our study was cross-sectional. Future studies can further investigate our findings in a larger group of subjects who can be followed longitudinally.

## Conclusions

In summary, our findings provide evidence that children with T1DM, despite their young age and a short duration of diabetes exhibit atherogenic lipoprotein profiles characterized by higher small HDL particles and higher LDL particles. Furthermore, children with T1DM show evidence of decreased HDL function and have lower CEC compared to healthy controls. In addition, the use of NMR appears to be superior to the standard lipid measurements to detect lipoprotein abnormalities in youth with T1DM as no differences in total HDL were detected with the use of the standard lipid profile between T1DM and HC groups. Also, given that low CEC is associated with small and large HDL particles, we suggest that the size of HDL particles could be used as a surrogate marker for low CEC if our results are replicated in future larger and prospective studies. Should our findings be confirmed, we would suggest the consideration of adding lipoprotein particles and cholesterol efflux in CVD risk stratification of youth with T1DM.

## Abbreviations

ABCA1: ATP-binding cassette transporter A1
BMI: Body Mass Index
CVD: cardiovascular disease
CIMT: Carotid Intima Media Thickness
CNHS: Children’s National Health System
FPLC: fast protein liquid chromatography
GU: Georgetown University
GHUCCTS: Georgetown-Howard Universities Center for Clinical and Translational Science
CEC: HDL-cholesterol efflux capacity
HC: healthy controls
HU: Howard University
IRB: Institutional Review Board
NMR: nuclear magnetic resonance
sHDLp: small size HDL particles
sLDLp: small size LDL particles
LDLp: total LDL particles
T1DM: type 1 diabetes
VLDLp: VLDL particles
